# Functional analysis of the first complete genome sequence of a multidrug resistant sequence type 2 *Staphylococcus epidermidis*

**DOI:** 10.1099/mgen.0.000077

**Published:** 2016-09-20

**Authors:** Jean Y. H. Lee, Ian R. Monk, Sacha J. Pidot, Siddarth Singh, Kyra Y. L. Chua, Torsten Seemann, Timothy P. Stinear, Benjamin P. Howden

**Affiliations:** ^1^​ Department of Microbiology & Immunology at The Doherty Institute for Infection & Immunity, University of Melbourne, Melbourne, Australia; ^2^​ Pacific Biosciences, Menlo Park, California, USA; ^3^​ Microbiology Department, Austin Health, Melbourne, Australia; ^4^​ Doherty Applied Microbial Genomics, Department of Microbiology & Immunology at The Doherty Institute for Infection & Immunity, University of Melbourne, Melbourne, Australia; ^5^​ Victorian Life Sciences Computation Inititative, University of Melbourne, Melbourne, Victoria, Australia; ^6^​ Microbiological Diagnostic Unit Public Health Laboratory, Department of Microbiology & Immunology at The Doherty Institute for Infection & Immunity, University of Melbourne, Melbourne, Australia; ^7^​ Infectious Diseases Department, Austin Health, Melbourne, Australia

**Keywords:** *Staphylococcus epidermidis*, antibiotic resistance, comparative genomics, methylome

## Abstract

*Staphylococcus epidermidis* is a significant opportunistic pathogen of humans. The ST2 lineage is frequently multidrug-resistant and accounts for most of the clinical disease worldwide. However, there are no publically available, closed ST2 genomes and pathogenesis studies have not focused on these strains. We report the complete genome and methylome of BPH0662, a multidrug-resistant, hospital-adapted, ST2 *S. epidermidis*, and describe the correlation between resistome and phenotype, as well as demonstrate its relationship to publically available, international ST2 isolates. Furthermore, we delineate the methylome determined by the two type I restriction modification systems present in BPH0662 through heterologous expression in *Escherichia coli*, allowing the assignment of each system to its corresponding target recognition motif. As the first, to our knowledge, complete ST2 *S. epidermidis* genome, BPH0662 provides a valuable reference for future genomic studies of this clinically relevant lineage. Defining the methylome and the construction of these *E. coli* hosts provides the foundation for the development of molecular tools to bypass restriction modification systems in this lineage that has hitherto proven intractable.

## Data Summary

All raw sequence data (reads and assembled genomes) for the *S. epidermidis* genomes analysed in this publication are publically available in the European Nucleotide Archive (ENA) under study accession numbers PRJEB12090 and PRJEB13975.

http://www.ebi.ac.uk/ena/data/view/PRJEB12090

1. *S. epidermidis* BPH0662 Illumina reads (ERS1019848)
  http://www.ebi.ac.uk/ena/data/view/ERS1019848
2. *S. epidermidis* BPH0663 Illumina reads (ERS1019849)
  http://www.ebi.ac.uk/ena/data/view/ERS1019849
  http://www.ebi.ac.uk/ena/data/view/PRJEB13975
3. *S. epidermidis* BPH0662 PacBio reads (ERS1153932)
  http://www.ebi.ac.uk/ena/data/view/ERS1153932
4. *E. coli* DC10B-MS1 PacBio reads (ERS1153933)
  http://www.ebi.ac.uk/ena/data/view/ERS1153933
5. *E. coli* DC10B-MS2 PacBio reads (ERS1153934)
  http://www.ebi.ac.uk/ena/data/view/ERS1153934
6. *S. epidermidis* BPH0662 complete genome assembly (GCA_900086615.1)
  http://www.ebi.ac.uk/ena/data/view/GCA_900086615

## Impact Statement

*Staphylococcus epidermidis* is a major nosocomial pathogen in humans, responsible for a significant proportion of implant- and device-associated infections that globally pose a significant burden on health-care systems. One multilocus sequence type, ST2, which is often multidrug-resistant, dominates *S. epidermidis* hospital-associated infections. However, the genetic structure and content of this clone are poorly understood. Additionally, because of limited understanding of the genetic barriers to the transformation of ST2 isolates, no molecular tools are available to manipulate and study this clone. Through in-depth genomic analysis of ST2 *S. epidermidis* we provide important insights into the genome structure, resistance mechanisms and barriers to DNA transformation and their relationship to other *S. epidermidis* clones. The generation of *E. coli* strains expressing ST2 *S. epidermidis* adenine methylation profiles provides the basis for the development of tools to genetically manipulate this globally dominant clone in the laboratory.

## Introduction

*Staphylococcus epidermidis* is a significant nosocomial pathogen, particularly in the setting of high-acuity medicine and prosthetic devices, where the presence of a foreign body provides a platform for bacterial colonisation. Identified by the Centers for Disease Control and Prevention as the leading cause of central-line-associated bloodstream infections, second ranked cause of surgical site infections and third most reported pathogen for hospital-acquired infections (Sievert *et al.*, 2013), *S. epidermidis* and other coagulase-negative staphylococci (CoNS) are estimated to cost $2 billion a year in the USA alone (Otto, 2009). Despite this significant burden imposed on healthcare systems, relatively little is understood about the mechanisms of pathogenesis and optimal treatment of *S. epidermidis*, with many assumptions extrapolated from *Staphylococcus aureus-*based studies.

*S. epidermidis* is the most genetically diverse species within the genus *Staphylococcus* (Becker *et al.*, 2014), with diversity also reported within the colonising strains of individual human hosts (Conlan *et al.*, 2012). In spite of this, hospital-based investigations utilising pulsed-field gel electrophoresis or multilocus sequence typing (MLST) typically demonstrate the predominance of one or two multidrug-resistant clones of *S. epidermidis* within the institutions studied (Gordon *et al.*, 2012; Krediet *et al.*, 2004; Widerström *et al.*, 2012). A single lineage, clonal complex 2, for which ST2 is the founder, accounts for 74 % of nosocomial isolates internationally (Miragaia *et al.*, 2007). The global dissemination of the ST2 lineage suggests its successful adaptation to the hospital environment in which favourable circumstances enable opportunistic infections. Knowledge of the genomics of ST2 strains has been limited by the lack of a closed ST2 reference genome, and the inability to genetically manipulate this lineage. The dearth of dedicated molecular tools and tractable clinical *S. epidermidis* strains has meant that pathogenesis studies have been performed on isolates that do not represent this clinically relevant clone that dominates worldwide.

The proportion of methicillin resistance in *S. epidermidis* has been reported to be as high as 92 % in some institutions (Krediet *et al.*, 2004), and is frequently associated with co-resistance to other antibiotic classes (Conlan *et al.*, 2012; Mendes *et al.*, 2012). Due to limited treatment options, vancomycin, a glycopeptide antibiotic considered one of the last-line agents for the treatment of staphylococci, is often utilised for serious infections.

The phenomenon of vancomycin intermediate heteroresistance is well described in *S. aureus* and is characterised by the presence of bacterial subpopulations capable of growth within the intermediate range despite testing as vancomycin-susceptible by standard laboratory methods. Such isolates are recognised as precursors to vancomycin intermediate *S. aureus* (VISA) and are associated with treatment failure (Tenover & Moellering, 2007). Unlike *S. aureus*, the definition and clinical implications of heterogenous vancomycin resistance in *S. epidermidis* is poorly understood. A limited number of studies have described the phenomena in *S. epidermidis* specifically (Gazzola *et al.*, 2013; Sieradzki *et al.*, 1999) or CoNS in general (Ma *et al.*, 2011; Nakipoglu *et al.*, 2005; Sieradzki *et al.*, 1998), however the mechanisms behind this resistance are unknown.

Restriction–modification (RM) systems are proposed to have evolved as a form of bacterial immunity that targets and degrades incoming DNA from viruses and other foreign donors (Casadesús & Low, 2006). Type I and type IV RM systems in particular, present strong barriers to the exchange of DNA in both *S. epidermidis* and *S. aureus* (Monk *et al.*, 2012). Type I RM systems are comprised of three host specificity of DNA (*hsd*) genes, that encode a specificity protein (HsdS), a modification protein (HsdM) and a restriction endonuclease (HsdR). The presence of adenine methylation on DNA sequences, determined by HsdS, termed target recognition motifs (TRMs), inhibits the restriction endonuclease complex. Unless methylated at appropriate sites, DNA is recognised as foreign and restricted (Murray, 2000). The type IV RM system of *S. epidermidis* consists of a single restriction endonuclease (formerly *mcrR*, now renamed *SepRPI*) that recognises cytosine-methylated DNA (Monk *et al.*, 2012). Plasmid artificial modification (PAM) is a means to overcome the barrier imposed by these RM systems, whereby vectors are constructed to express *hsdMS* genes that mimic the DNA methylation pattern of the bacterium into which they are transformed. A method of PAM in which *hsdMS* genes from *S. aureus* strains of interest were chromosomally integrated into a DC10B *E. coli* background, deficient of cytosine methylation, was recently described (Monk *et al.*, 2015). Plasmids isolated from the resulting *E. coli* mutants were capable of bypassing both type I and type IV RM systems of *S. aureus* strains from which their *hsdMS* genes were cloned.

In 2012, a case of persistent, post-neurosurgical infection with a strain of *S. epidermidis* exhibiting multidrug resistance, manifesting as a cerebral abscess associated with an external ventricular drain (EVD), occurred at our institution. Other than a single intraoperative dose of prophylactic cephazolin, the patient had no prior history of antibiotic therapy or relevant hospitalisation. Resistant to commonly encountered agents including flucloxacillin, erythromycin, clindamycin and cotrimoxazole; it was of concern that the isolate also demonstrated resistance to rifampicin, fusidic acid, ciprofloxacin and teicoplanin (a glycopeptide). Extended glycopeptide-susceptibility testing performed in the context of failing vancomycin therapy, showed that the isolate was vancomycin heteroresistant, prompting investigations to further characterise this hospital adapted strain.

Here we use Pacific Biosciences single molecule real-time (SMRT) sequencing to describe the first complete ST2 *S. epidermidis* genome sequence and its methylome, and demonstrate its function as an improved reference for the bioinformatic analysis of international strains belonging to this clinically relevant lineage.

## Methods

### Media and reagents.

*S. epidermidis* and *S. aureus* were routinely cultured at 37 ^o^C in brain heart infusion (BHI) broth (Difco). *E. coli* was routinely cultured in L broth (1 % tryptone, 0.5 % yeast extract, 0.5 % NaCl). Broth microdilution (BMD) MICs were performed in cation-adjusted Mueller–Hinton (Difco) medium. For growth on agar, BHI or L broth were solidified with 1.5 % agar, to yield BHIA and L agar, respectively. The following antibiotics were purchased from Sigma Aldrich and used at the specified concentrations: chloramphenicol (Cm) 15 µg ml^−1^ in *E. coli*; ampicillin (Amp) 100 µg ml^−1^ in *E. coli*; kanamycin (Kan) 50 µg ml^−1^ in *E. coli.* The following antibiotics were used at variable concentrations for susceptibility testing: rifampicin (Rif) (Sigma Aldrich); vancomycin (Hospira).

Oligonucleotides were purchased from Integrated DNA Technologies and are listed in Table 1. Genomic DNA for routine use was isolated with the DNeasy Blood and Tissue Kit (Qiagen). To weaken the cell wall of *S. epidermidis* prior to DNA extraction, harvested cells were washed with PBS, lysostaphin (Ambi) was added to the Gram-positive lysis buffer (final concentration 100 µg ml^−1^) and incubated at 37 ^o^C for 30 min. Genomic DNA for SMRT sequencing was isolated using the Qiagen Genomic-tip 100 G (Qiagen). Plasmids were purified with a QIAprep Spin Miniprep Kit (Qiagen). PCR products and gel extractions were purified using a QIAquick Gel Extraction Kit (Qiagen). Restriction enzymes and Phusion DNA polymerase were purchased from New England Biolabs. Phire Hotstart DNA polymerase was purchased from Thermofisher. Colony PCR was performed as previously described (Monk *et al.*, 2012).

**Table 1.** Strains, plasmids and oligonucleotides used in this study

Bacterial strain, plasmid or oligonucleotide	Description	Reference or source
*E. coli* strains		
DC10B	DH10B with ∆*dcm* mutation	Monk *et al.* (2015)
DC10B-MS1	DC10B with SepiBPH0662I *hsdMS* integrated between *ybbD* and *ylbG*	This study
DC10B-MS2	DC10B with SepiBPH0662II *hsdMS* integrated between *essQ* and *cspB*	This study
*S. epidermidis* strains		
ATCC 12228	Non-clinical reference strain	Zhang *et al.*(2003)
BPH0662	Clinical strain. Index patient, isolated day 12	This study
BPH0663	Clinical strain. Index patient, isolated day 23	This study
*S. aureus* strains		
Mu3	Heteroresistant vancomycin intermediate *S. aureus* reference strain	Hiramatsu *et al.* (1997a)
Mu50	Vancomycin intermediate *S. aureus* reference strain	Hiramatsu *et al.* (1997b)
Plasmids		
pKD4	Plasmid for amplification of FRT-*kan*-FRT; Amp^R^, Kan^R^	Datsenko & Wanner (2000)
pKD46	*E. coli* temperature-sensitive plasmid containing λ red recombinase genes under the control of an arabinose-inducible promoter; Amp^R^	Datsenko & Wanner (2000)
pCP20	*E. coli* temperature-sensitive plasmid expressing *flp* enzyme for *flp*-catalysed excision of *kan* marker; Amp^R^, Cm^R^	Cherepanov & Wackernagel (1995)
Cloning *hsdMS* genes		
IM199	CCCAAACTGCACCCAAGAGTCAGAACACAGTTTTTCAAGAGTACAAAGGGGTAAACTAAAATAAATATTGACACTCTATCATTG	This study
IM200	AAACTAAAATAAATATTGACACTCTATCATTGATAGAGTATAATTAAAATAAGGAGGAAATTAATGGCAACTATTGGATTTGAAG	This study
IM201	TTAAATTGAAAGTTCATCACTATTCACCTC	This study
IM202	GGTGAATAGTGATGAACTTTCAATTTAAGTGTAGGCTGGAGCTGCTTC	This study
IM203	GCTAACCATTGTGGTGAAGTGCAGGTTTGCTGCATGAATAGTTTTACGGTCCATATGAATATCCTCCTTAG	This study
IM204	ACTGAGAAAAGACATGTCGGCTATTGTGTAAAGCCATATAGCTCAGACGACATAAAAAATTTATTTGCTTTCAGG	This study
IM205	CATAAAAAATTTATTTGCTTTCAGGAAAATTTTTCTGTATAATAGATTCATAAATTTGAGAGAGGAGTTATGTCAACGACGGAAAAACAAAGAC	This study
IM206	CTACACAAACATCTTCTGTAAAAAGCC	This study
IM207	GCTTTTTACAGAAGATGTTTGTGTAGGTGTAGGCTGGAGCTGCTTCG	This study
IM208	TTCTATGTAAACTCTCTGACTGTTCATTTTATTTGTTGTTTCAGGGTCGGGGTCCATATGAATATCCTCCTTAG	This study
Recombineering		
IM177	TCGAAGTCGTCAACTTCGTAGTGAGG	This study
IM178	TGCCGCTGGTTTTCCGCTAATGG	This study
IM179	CGGCCATTTATACAGGAAAAGCCTA	This study
IM180	GTTACCTTCTCTATAGAGAGTGGTG	This study

Amp^R^, ampicillin resistant; Kan^R^, kanamycin resistant; Cm^R^, chloramphenicol resistant

### Bacterial isolates.

Bacterial strains used in this study are listed in Table 1. Phenotypic comparator strains consisted of three reference strains: *S. epidermidis* ATCC 12228, *S. aureus* ATCC 700698 [Mu3] and ATCC 700699 [Mu50].

### Antibiotic susceptibility testing.

Vitek 2 (bioMerieux) susceptibilities were tested as per the manufacturer’s instructions. BMD MICs for vancomycin and rifampicin were determined as recommended by the Clinical and Laboratory Standards Institute (CLSI, 2012). Extended glycopeptide susceptibilities were determined by the macromethod Etest (MET) with vancomycin and teicoplanin Etest strips (bioMerieux) using a 2.0 McFarland inoculum and prolonged incubation time of 48 h; vancomycin population analysis profiles (PAPs) were performed as previously described (Wootton *et al.*, 2001). With the exception of Vitek 2 and Etests, all antibiotic-susceptibility testing of strains was performed in triplicate.

### Genome sequencing.

Sequencing of BPH0662 and BPH0663 was performed using the Illumina MiSeq platform with Nextera XT libraries constructed as per the manufacturer’s instructions. SMRT sequencing on a PacBio *RS* instrument with subsequent *de novo* assembly using HGAP2 algorithm, and base modification and motif detection for methylome analysis using SMRT Analysis v1.3.1 was performed for *S. epidermidis* BPH0662. *E. coli* mutants DC10B-MS1 and DC10B-MS2 were assembled and analysed as previously described (Monk *et al.*, 2015). RP62a was used as the methylation reference for BPH0662, while DH10B was used as a reference for the *E. coli* strains. Final assembly was validated by reference to a high-resolution *Nco*I chromosome optical map using MapSolver (v3.2.0, OpGen), as previously described (Gao *et al.*, 2015). BPH0662 Illumina reads were used to correct homopolymer errors in the PacBio assembled BPH0662 genome. BPH0662 Illumina reads were assembled using SPAdes v3.7.1 (Bankevich *et al.*, 2012) and the resulting contigs were screened for small plasmids that would be lost during PacBio size selection.

### Genome analysis.

Variant calling between the closed BPH0662 genome and BPH0663 Illumina reads was confirmed by two methods, using Snippy v3.0 (Seemann, 2016a) and Nesoni (Harrison, 2014). A one-way comparison of gene content between each of the four published, closed *S. epidermidis* genomes and BPH0662 was performed using Blast Ring Image Generator (BRIG) (Alikhan *et al.*, 2011). Annotated prophages in BPH0662 were identified using PHage Search Tool (PHAST) (Zhou *et al.*, 2011). The presence of CRISPRs was screened for with CRISPRfinder (Grissa *et al.*, 2007). *In silico* MLST was performed on the *de novo* assemblies (Seemann, 2016b).

All publically available, assembled *S. epidermidis* genomes in NCBI GenBank were downloaded and analysed (4th April 2016). Selection criteria for inclusion as a comparator strain were as follows: *in silico* MLST categorisation as ST2. The exclusion criteria were: sequencing performed on a Roche 454 or Ion Torrent platform (per GenBank metadata); Illumina sequencing with depth <25× (per GenBank metadata); assembled genome size >120 % of the BPH0662 genome (surrogate marker for mixed bacterial samples); organism not *S. epidermidis* determined by Kraken v0.10.5beta (Wood & Salzberg, 2014). This resulted in selection of 32 potential comparator genomes from 307 assemblies. For isolates where core-SNP phylogeny of the 32 strains indicated clonal groupings, a single representative isolate from each group was included. The resulting 15 unique comparator genomes with metadata are listed in Table S1 (available in the online Supplementary Material).

Genome annotation was performed with Prokka v1.12 (Seemann, 2014). Maximum-likelihood core-SNP phylogeny of *S. epidermidis* isolates using the newly closed BPH0662 genome as a reference, was determined using Snippy v3.0 (Seemann, 2016a) to generate an alignment of core SNPs, PhyML v3.1 (Guindon *et al.*, 2010) was then used to reconstruct a maximum-likelihood tree. Pairwise SNP analysis was performed using pairwise_snp_differences (Goncalves da Silva, 2015). For pangenome analysis, protein ortholog clustering was performed using Proteinortho v5.11(Lechner *et al.*, 2011), alignment of the resultant CDS orthologs to BPH0662 and visualisation of the pangenome was performed using FriPan (Powell, 2015).

### Construction of *E. coli* DC10B-MS1 and DC10B-MS2.

*E. coli* mutants expressing the BPH0662 type I RM systems in a DC10B background were created using the method previously described by Monk *et al.* (2015). *E. coli* DC10B-MS1, which expressed SepiBPH0662I *hsdMS (hsdMS1*) was constructed as follows (Fig. S1). The *hsdMS1* system from BPH0662 was amplified with primers IM205 (incorporating PN25 promoter)/IM206. Plasmid pKD4 (Datsenko & Wanner, 2000) was used as a template for the amplification of the Kan resistance marker flanked by flippase recognition target (FRT) sites with primers IM207/IM208 (product pKD4-1). The hsdMS1 and pKD4-1 products were gel-extracted and joined by spliced overlap extension (SOE) PCR using primers IM204/208 (primer tails contained 50 bp homology for integration into the intergenic region between *ybbD* and *ylbG* in the DC10B chromosome). The hsdMS1–pKD4-1 linear amplicon was pellet paint (Novagen) precipitated and electroporated into DC10B containing pKD46 (Datsenko & Wanner, 2000), made competent as previously described (Monk *et al.*, 2012). Transformants were selected for on L agar Cm10. Colony PCR with primers IM177/IM178 was used to screen for recombination at the integration site, positive clones were grown overnight at 43 ^o^C to promote loss of pKD46. To excise the pKD4-1 product, strains were transformed with pCP20 (Cherepanov & Wackernagel, 1995) at 28 ^o^C, single-colony purified at 28 ^o^C, then grown overnight at 43 ^o^C to promote plasmid loss. To confirm excision of the Kan resistance marker and the loss of pCP20, cells were patch plated on L agar Kan and L agar Cm, respectively. The resulting Kan^S^, Cm^S^ strain containing *hsdMS1* (with PN25 promoter) was named DC10B-MS1.

Using the same protocol, the *E. coli* DC10B-MS2 mutant was constructed from *E. coli* DC10B using the following primer sets: IM200 (incorporating Pxyl/tetO promoter)/IM201 for the amplification of SepiBPH0662II *hsdMS (hsdMS2*); IM202/IM203 for amplification of FRT-*kan*-FRT from pKD4 (product named pKD4-2); IM199/IM203 (containing tails with 50 bp homology for integration into the intergenic region between *essQ* and *cspB* in the DC10B chromosome) for SOE PCR of hsdMS2-pKD4-2; IM179/IM180 to confirm recombination at integration site.

## Results & Discussion

### Identification of a clinical multidrug-resistant *S. epidermidis* displaying vancomycin heteroresistance

**Fig. 1.**
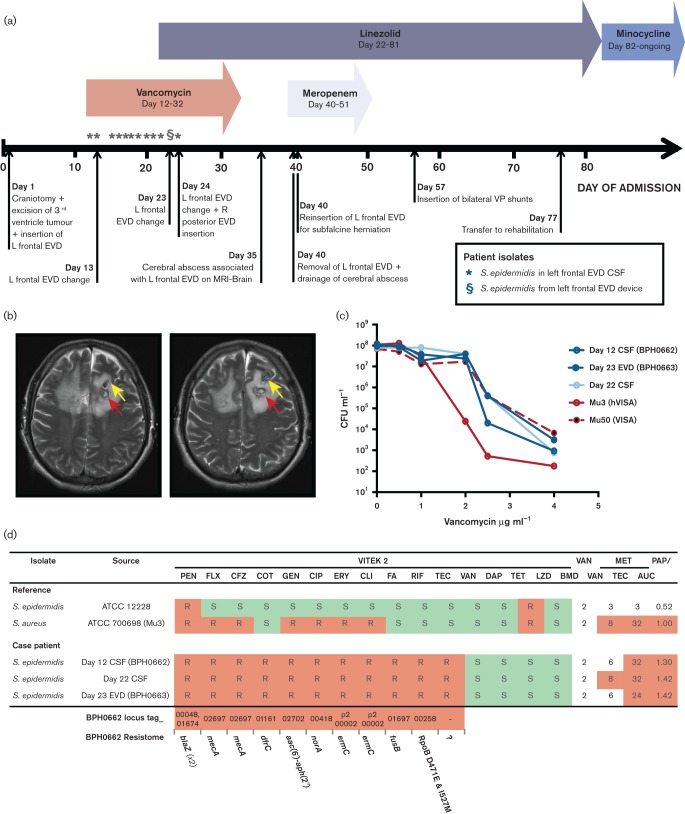
BPH0662 - a clinically significant, multidrug-resistant *S. epidermidis.* (a) Summary of case patient’s clinical course. (b) Magnetic resonance imaging (MRI) slices of the patient’s brain, demonstrating a large cerebral abscess (red arrow) formed around the tip of an external ventricular drain (EVD) device (yellow arrow). (c) Vancomycin population analysis profile of three *S. epidermidis* isolates from the case patient compared with reference strains Mu3 [heterogeneous vancomycin intermediate *S. aureus* (hVISA)] and Mu50 [vancomycin intermediate *S. aureus* (VISA)]. (d) Correlation of phenotypic susceptibility testing for case patient isolates with the BPH0662 resistome. L, left; R, right; VP, ventriculoperitoneal; CSF, cerebrospinal fluid; PEN, penicillin; FLX, flucloxacillin; COT, cotrimixazole; GEN, gentamicin; CIP, ciprofloxacin; ERY, erythromycin; CLI, clindamycin; FA, fusidic acid; RIF, rifampicin; TEC, tecoplanin; VAN, vancomycin; DAP, daptomycin; TET, tetracycline; LZD, linezolid; BMD, broth microdilution; MET, macromethod Etest; PAP/AUC, population analysis profile/ area under the curve (compared with Mu3 hVISA reference).

While multidrug resistance in clinical isolates of *S. epidermidis* is increasingly common, standard antimicrobial susceptibility testing demonstrated BPH0662 was more broadly resistant than usually reported (resistant to rifampicin, fusidic acid and teicoplanin). Despite testing as susceptible to vancomycin with a MIC of 2 µg ml^−1^ using the gold standard method of BMD, the patient failed vancomycin therapy (Fig. 1d). Phenotypically identical *S. epidermidis* were repeatedly isolated from both EVD cerebrospinal fluid (CSF) and sterile surgical site samples from the patient, on 12 occasions over 13 days (Fig. 1a, d). To investigate the mechanism of treatment failure, extended glycopeptide susceptibility testing using vancomycin and teicoplanin METs and vancomycin PAPs was performed. Although no diagnostic criteria has been defined for the classification of vancomycin heteroresistance in *S. epidermidis*, if the criteria used for *S. aureus* were applied (≥8 µg ml^−1^ for both vancomycin and teicoplanin MET, or ≥12 µg ml^−1^ for teicoplanin MET regardless of vancomycin MET; PAP:AUC of ≥0.9 compared with Mu3 standard for vancomycin PAP) then all of the patient’s *S. epidermidis* isolates were found to be vancomycin-heteroresistant by both testing methods (Fig. 1c, d). This demonstrated that the true antibiogram of BPH0662 was more complex than initially appreciated, with the isolates approaching pan-drug resistance. Vancomycin is a last-line agent in the treatment of staphylococci, therefore evolution of resistance to this antimicrobial left no conventional therapeutic options. In spite of this, the patient was successfully treated with a combination of surgical source control and change in therapy to linezolid, followed by life-long suppressive minocycline therapy.

### Establishing a complete ST2 reference genome

**Fig. 2.**
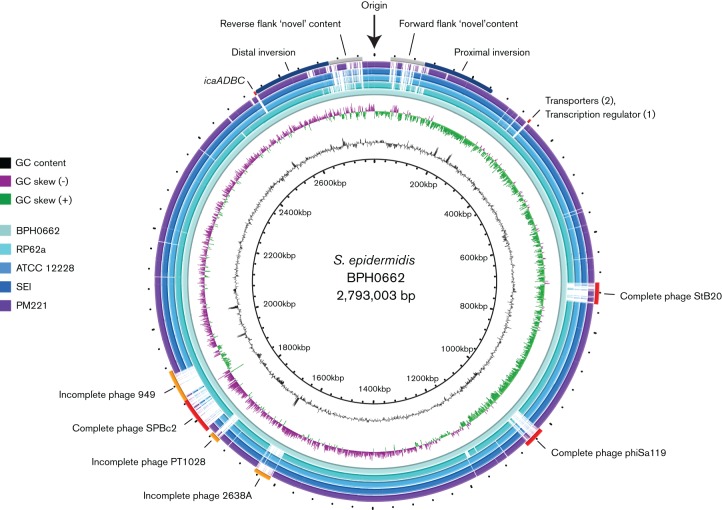
Hospital-evolved strain BPH0662 possesses novel gene content acquired through horizontal gene transfer. A one-way comparison of gene content between the four published *S. epidermidis* genomes and BPH0662 was generated using BRIG (Alikhan *et al.*, 2011). Prophages are annotated in the outermost ring.

**Fig. 3.**
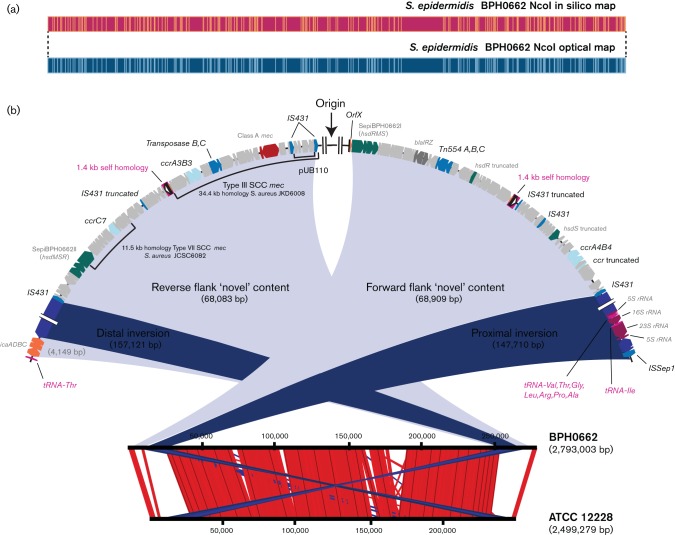
The novel structure of *S. epidermidis* BPH0662 with unusual location of a chimeric SCC*mec*. (a) *In silico* NcoI map of the BPH0662 genome assembly aligned with NcoI optical map of BPH0662 genome. (b) The closed genome of BPH0662 aligned to the *S. epidermidis* ATCC 12228 reference genome, demonstrating the inversion associated with the unusual location and orientation of the SCC*mec* element in BPH0662.

To confirm that these isolates represented persistent infection with the same strain and to further investigate the molecular characteristics underlying the near pan-resistant phenotype of these isolates, we initially performed Illumina whole-genome sequencing of BPH0662 (initial CSF isolate, day 12 of admission) and BPH0663 (intraoperative isolate from EVD device, day 23). Analysis indicated that both were ST2 *S. epidermidis*. Despite being the globally dominant hospital lineage, no complete reference sequence of an ST2 *S. epidermidis* strain was available. Therefore, the genome of BPH0662 was analysed using SMRT sequencing (Fig. 2), and validated by comparison to an optical map generated for the same isolate (Fig. 3a). The *S. epidermidis* BPH0662 genome comprises a 2 793 003 bp circular chromosome with 32.0 % DNA G+C content, and three circular plasmids. The largest plasmid (pBPH0662-01) is 45 807 bp with 30.0 % DNA G+C content, the second (pBPH0662-02) only 2366 bp with 31.1 % DNA G+C content, and the third (pBPH0662-03) is 13 569 bp with 28.6 % DNA G+C content. The chromosome was predicted to contain 2653 coding genes, with an additional 40 genes located on pBPH0662-01. Containing only two coding regions, *repL* and *ermC*, pBPH0662-02 shared 99 % DNA sequence similarity with the naturally occurring, 2355 bp plasmid pNE131 (Genbank M12730.1) previously described in clinical *S. epidermidis* strains (Lampson & Parisi, 1986a). Notably, pBPH0662-02 possesses the 107 bp deletion in the 5′ regulatory region of *ermC* to which the constitutive expression of macrolide–lincosamide–streptogramin B resistance in pNE131 has been attributed (Lampson & Parisi, 1986b). Furthermore, plasmids genetically identical (Genbank AB982225.1) to pBPH0662-02 have been described in Russian, ST8, community-acquired methicillin-resistant *S. aureus* strains (Khokhlova *et al.*, 2015). A further 14 predicted coding genes were on pBPH0662-03, including the antiseptic resistance gene *qacA* and its regulator. Analysis of the Illumina-sequenced BPH0663 genome compared with the closed BPH0662 genome indicated that the isolates were clonal, with no detectable SNPs between them.

Comparisons between BPH0662 and the four existing, complete, *S. epidermidis* genomes are shown in (Fig. 2) and Table 2. Similar to the biofilm-forming, drug-resistant clinical strain RP62a (Gill *et al.*, 2005), BPH0662 possessed the *icaADBC* operon and *mecA*. Analysis indicated that the majority of novel gene content in BPH0662 was acquired by horizontal gene transfer. Three complete and three partial prophages were detected, accounting for 9.3 % of the genome. Further novel content was concentrated in the regions flanking the origin of replication, which contained multiple resistance determinants, drug transporters and regulatory genes, interspersed between numerous insertion-sequence (IS) elements (particularly IS*431*) and transposases. No clustered regularly interspaced short palindromic repeat (CRISPR) loci were identified in the genome. The phenotypic susceptibility testing of BPH0662 correlated with the resistome is shown in (Fig. 1d). Genetic determinants accounting for the phenotypic resistance observed for BPH0662 were identified for all antimicrobial agents with the exception of teicoplanin and vancomycin, both glycopeptide antibiotics. In the related species *S. aureus*, rifampicin-resistance mutations in *rpoB* have been demonstrated to result in vancomycin heteroresistance (Gao *et al.*, 2013; Matsuo *et al.*, 2011), leading us to speculate that the same phenomenon may also occur in *S. epidermidis*. Dual D471E and I527M mutations were identified in the BPH0662 RpoB, associated with rifampicin resistance (MIC of 64 µg ml^−1^). The same dual substitutions have previously been described as the RpoB mutations most commonly associated with high-level rifampicin resistance in a *S. epidermidis* prosthetic joint infection study (Hellmark *et al.*, 2009).

**Table 2.** Comparison of completed *S. epidermidis* genomes

Strain	Chromosome (Mb)	Plasmids	GC %	Genes	Source	*In silico* MLST	Reference
BPH0662	2.79	3	32.0	2709	Clinical	2	This study
RP62a/ ATCC 35984	2.61	1	32.2	2662	Clinical	10	Gill *et al.* (2005)
SEI/ ATCC 49134	2.50	1	32.0	2397	Non-clinical	*-	Zhang *et al.* (2012)
ATCC 12228	2.50	6	32.1	2558	Non-clinical	8	Zhang *et al.* (2003)
PM221	2.49	4	31.9	2461	Bovine mastitis	184	Savijoki *et al.* (2014)

*Published reference strain SEI was not classifiable by existing multi-locus sequence type (MLST) schema.

BPH0662 contained an unusual variant of staphylococcal cassette chromosome *mec* (SCC*mec*) type III, as it was located in reverse orientation in the region upstream of the origin of replication (Fig. 3b). The element was not associated with the typical integration site *orfX* [corresponding to the last 15 nucleotides of the coding sequence of the rRNA large subunit methytransferase (Boundy *et al.*, 2013)], located approximately 32 kb downstream from the origin of replication in BPH0662. Despite the unexpected location on the chromosome, the BPH0662 SCC*mec* element contained *ccrA3B3* and a prototypical class A *mec* complex, with 34.4 kb of the element sharing 99.8 % nucleotide homology with the previously described, 37 kb type III SCC*mec* of ST239 *S. aureus* strains JKD6008 (GenBank CP0021020) and Sa0059 (GenBank JQ412578), including the integrated plasmid pUB110 (harbouring genes encoding kanamycin and bleomycin resistance). Based on the *orfX* sequence in BPH0662, a perfect inverted repeat, and imperfect direct repeat, were present in the distal (upstream) end of the SCC*mec* element in reverse orientation, in keeping with the reverse orientation of the entire element (Fig. S2), suggesting initial integration of the SCC*mec* element at *orfX* with subsequent chromosomal rearrangement. Two 1.4 kb regions of DNA homology potentially explained this rearrangement. These regions mirrored one another on either side of the origin of replication: one located 59.7 kb downstream; the other 71.8 kb at the reverse flank, which incorporated the upstream direct repeat of the SCC*mec* element (Fig. 3b).

Located 20 kb upstream of SCC*mec* was a *ccrC7* in close proximity to a complete type I RM, flanked at both ends by IS*431* [also known as IS*257* (Rouch & Skurray, 1989)] elements. The carriage of *hsdRMS* genes in association with *ccrC* is described in both type V and VII SCC*mec* in *S. aureus* [Berglund *et al.*, 2008; International Working Group on the Classification of Staphylococcal Cassette Chromosome Elements (IWG-SCC), 2009]. Indeed, 11.5 kb of this region in BPH0662, encompassing *ccrC7* extending into *hsdR2,* displayed 98 % homology with joining regions 2 and 3 and the *ccr* gene complex of the prototypical, type VII SCC*mec* described in *S. aureus* strain JCSC6082 (GenBank AB373032). Forward of the origin of replication, another chimeric SCC element was present, containing a *ccrA4B4* complex as well as one complete and a truncated type I RM system, in the absence of a *mec* complex (see Fig. 3b). Remnants of an additional truncated *ccr* gene were in close proximity.

The assortment of *ccr* genes observed in BPH0662 was in keeping with the reported diversity in coagulase-negative staphylococci (Hanssen & Sollid, 2007), suggested to be the donor species in which novel and composite SCC elements arise and are then transferred to the more genetically conserved *S. aureus* (Barbier *et al.*, 2010). The presence of a near complete type III SCC*mec* in BPH0662, identical to that previously described in ST239 *S. aureus* [a globally disseminated, healthcare-associated, clonal lineage of methicillin-resistant *S. aureus* (Harris *et al.*, 2010)] reinforces the probable role of these recombinases in the horizontal transfer of DNA between staphylococcal species.

Compared with the closed reference genome of *S. epidermidis* ATCC 12228, the overall structure of BPH0662 was suggestive of chromosomal rearrangement resulting from two inversions around the origin of replication (Fig. 3b). The inversion points closest to the origin, potentially attributable to homologous recombination between the regions of self-homology as described above, and the medial inversions, possibly explained by the nearby presence of tRNA genes, which have been demonstrated to be associated with chromosomal inversions in other bacterial species (DeBoy *et al.*, 2006). Relative to ATCC 12228, RP62a demonstrates a single inversion around the origin of replication (Gill *et al.*, 2005; Lindsay & Holden, 2007), corresponding to the most medial inversions observed in BPH0662 (with a series of tRNA genes and an adjacent ISSep element at one inversion point, and a lone tRNA-Thr adjacent to the *icaADBC* operon at the other). Large-scale chromosomal inversions and deletions near the origin of replication have also been described in other CoNS, associated with homologous recombination of IS elements (Watanabe *et al.*, 2007). While this arrangement around the origin with a double inversion is unusual, the chromosome assembly of BPH0662 was confirmed to be correct by optical mapping (Fig. 3a). The importance of independent verification of genome assembly for the identification of large chromosomal rearrangements associated with biological consequences has recently been described (Gao *et al.*, 2015). Furthermore, the value of a closely related reference genome for comparative genomics is becoming increasingly evident (Pightling *et al.*, 2014). In view of the global dominance of the ST2 lineage within the hospital environment, as the first, to our knowledge, completed ST2 genome, BPH0662 will be invaluable for future genomic studies of clinically relevant *S. epidermidis* isolates.

### The BPH0662 methylome

**Fig. 4.**
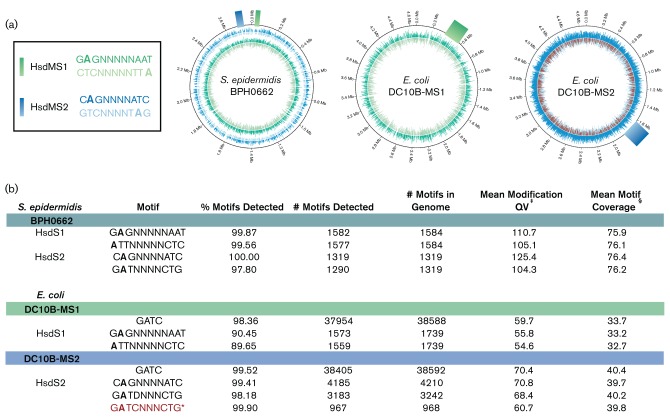
The type I restriction modification (RM) systems of BPH0662. (a) Two functional type I RM systems, encoding SepiBPH0662I (HsdMS1) and SepiBPH0662II (HsdMS2), were identified in the *S. epidermidis* BPH0662 genome. *E. coli* hosts expressing HsdMS1 (DC10B-MS1) and HsdMS2 (DC10B-MS2) were engineered and SMRT sequencing was used to determine their methylomes, allowing correlation of the BPH0662 target recognition domains (TRDs) with their corresponding HsdMS systems. The chromosomal position of each HsdMS system and its associated methylated adenine residues (represented by a line whose length corresponds with the interpulse duration of the read) for BPH0662 and the two *E. coli* host strains were plotted using Circos (Krzywinski *et al.*, 2009). (b) Using the SMRT Suite Pipeline v2.2.0/Motif Finder v1.3.1 to analyse reads, the conserved adenine-methylated residues (in bold type) and TRMs associated with the HsdS alleles of *S. epidermidis* BPH0662 and the *E. coli* host strains DC10B-MS1 and DC10B-MS2 were identified. ^†^Mean modification QV is defined as the quality value of the base calls within the motif; ^‡^Mean motif coverage is defined as the average depth of read coverage within a motif; *The HsdMS2 TRM (GATCNNNCTG) overlapped with endogenous *dam* (GATC) methylation in the *E. coli* host.

The pattern of adenine-methylated DNA detected by SMRT sequencing of BPH0662 indicated the presence of two functional type I RM systems, corresponding with two independent TRMs (Fig. 4a), formally named SepiBPH0662I and SepiBPH0662II, as per standard nomenclature (Roberts *et al.*, 2003). An incomplete third system with a predicted non-functional partial *hsdS* was noted in the genome. To determine the assignment of each *hsdMS* system with its associated TRM, two DC10B-derived *E. coli* hosts were constructed, each containing an individual *hsdMS* system from BPH0662 (DC10B-MS1 and DC10B-MS2). Integration of the BPH0662 *hsdMS* systems into DC10B at the expected sites was confirmed by SMRT sequencing of DC10B-MS1 and DC10B-MS2. Functional expression of the systems by the *E. coli* hosts was demonstrated by the acquisition of chromosomal adenine methylation at a high proportion (89.65–99.90 %) of predicted target recognition domains (TRDs) determined by the integrated BPH0662 *hsdS* (Fig. 4b). Notably, the 5′-G**A**TNNNNCTG-3′ TRM of DC10B-MS2 was predicted to overlap with endogenous *dam* (5′-G**A**TC-3′) methylation at 968 sites in the *E. coli* genome, SMRT sequencing indicated that 967 of these sites were appropriately methylated. Construction of these two *E. coli* hosts sets the groundwork for future experiments where a second integration event (either SepiBPH0662II *hsdMS* into DC10B-MS1 or SepiBPH0662I *hsdMS* into DC10B-MS2), resulting in a single *E. coli* host expressing both BPH0662 type I RM systems, should enable utilisation of plasmid artificial modification to overcome the strong restriction barrier present in *S. epidermidis* isolates from the ST2 lineage, facilitating the genetic manipulation of these clinically relevant clones that have hitherto proven refractory.

### BPH0662 in relation to international ST2 *S. epidermidis*

**Fig. 5.**
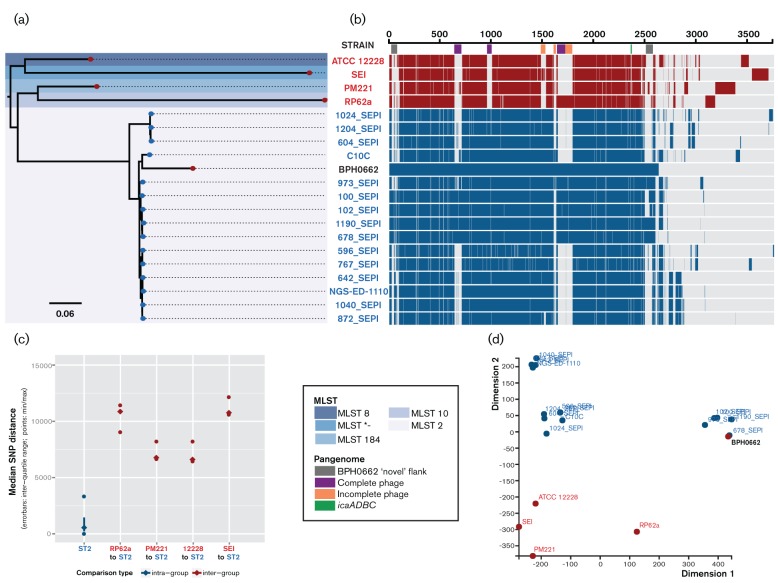
Comparative genomics of BPH0662 in relation to existing sequenced genomes and published international ST2 *S. epidermidis* isolates. (a) Maximum-likelihood, core-SNP-based phylogeny of 15 representative international ST2 isolates, the four existing sequenced strains and the new BPH0662 ST2 reference strain (scale bar indicates nucleotide substitutions per site); aligned with (b) Pangenome. (c) Intra-group comparison of pairwise core-SNPs for BPH0662 together with the international ST2 strains; and inter-group comparison of pairwise core-SNPs for the existing references, compared with the ST2 strains. (d) Multidimensional scaling of pangenome coding sequence orthologs. *Published reference strain SEI was not classifiable by existing multi-locus sequence type (MLST) schema.

At the time of analysis, 307 partially assembled *S. epidermidis* genomes were publically available from NCBI Genbank. *In silico* MLST identified 87 of these as ST2 isolates. Due to the large number of genomes that were sequenced as part of microbiome projects, a large proportion had poor sequencing coverage and/or contained reads mixed with other species, both of which resulted in questionable assemblies. Furthermore, some genomes represented other bacterial species incorrectly classified as *S. epidermidis*. Using the outlined selection/exclusion criteria, a curated list of 15 genomes from three different projects [two published (Roach *et al.*, 2015; Walsh *et al.*, 2015), one unpublished PRJNA246628] was obtained (Table S1). To determine how the newly closed BPH0662 isolate related to international ST2 strains, analysis of these 15 representative, publically available, draft ST2 genomes together with the four existing complete *S. epidermidis* genomes was performed using BPH0662 as a reference (Fig. 5a). A maximum-likelihood core-SNP-based phylogeny for these 20 strains was performed, aligned with their respective complete coding sequence content (pangenome analysis) (Fig. 5b). Phylogenetically, the BPH0662 reference sits within the characterised, international ST2 *S. epidermidis* clade, with an observed 559 median SNPs difference within the ST2 intragroup (Fig. 5c). The *icaADBC* operon was observed to be present in all ST2 strains and RP62a, but none of the non-human-host reference strains (Fig. 5b). Methicillin-resistance determinant *mecA*, was present in 14 of the 15 ST2 clinical comparators, with the exception of 642_SEPI.

Interestingly, five of the 15 international ST2 isolates grouped closely to BPH0662 when coding sequence ortholog presence/absence was analysed by multidimensional scaling (Fig. 5d). While the exact structures of these isolates could not be determined from their draft assemblies, detailed analyses of contigs of interest revealed that all five possessed over 27.5 kb of the BPH0662 type III SCC*mec* core region, spanning the class A *mec*, J2 region and *ccrA3B3* (Fig. S2c). Due to contig breaks corresponding with an IS*431* insertion sequence in the BPH0662 SCC*mec*, it is not known whether these five isolates also possessed the same J3 region, with an integrated pUB110. However, all but one (102_SEPI) contained pUB110 as a single contig. Intriguingly, the J1 region of three of the isolates (1190_SEPI, 678_SEPI and 102_SEPI) spanned 15 kb containing the full SepiBPH0662I type I RM system with identical downstream genes to BPH0662 (Fig. S2a, c). Furthermore, two of these isolates (1190_SEPI and 678_SEPI) also possessed a 26 kb contig composed of the SepiBPH0662II system associated with *ccrC7,* with identical intervening gene arrangement as BPH0662 (Fig. S2c). A single isolate, 100_SEPI, possessed only the SepiBPH0662I system, in the same position as BPH0662, adjacent to *orfX*. While 973_SEPI contained only SepiBPH0662II (Fig. S2c). Despite similar total content, the composite structure of these ST2 comparators highlights the role of mobile elements in the translocation and horizontal transfer of novel gene clusters.

Like BPH0662, these five ST2 isolates were all collected from critically ill patients in intensive care (Roach *et al.*, 2015). In view of the gene content common to these strains, including resistance determinants such *mecA*, *aadD* and *aac(6′)-aph(2″)* together with the same type I RM systems, and the circumstances in which they were collected, it could be proposed that BPH0662 and the closely clustered isolates may represent a successful hospital-adapted sublineage of ST2. This reinforces the relevance of BPH0662 as a representative ST2 strain and the potential applicability of the constructed *E. coli* hosts for manipulation of international ST2 *S. epidermidis* isolates.

Notably, RP62a, a ST10 strain frequently utilised as a reference isolate in *S. epidermidis* studies was observed to be particularly divergent from ST2 isolates based on median pairwise SNP difference, with a predicted 10 8612 SNPs between the groups (Fig. 5c). Furthermore, shared ortholog clustering indicated that RP62a and the other three existing reference genomes are divergent from ST2 isolates (Fig. 5d), overall indicating that RP62a may not be the most appropriate reference strain for studies of clinically relevant phenotypes.

## Conclusions

Evolving within the hospital environment, analysis of the BPH0662 genome indicates that this isolate has undergone chromosomal rearrangements and multiple horizontal gene transfer events resulting in the accumulation of a broad range of resistance determinants enabling its establishment as a successful multidrug-resistant hospital clone. Furthermore, the likely role of *S. epidermidis* as a potential donor species for the generation and transfer of resistance elements to other staphylococci is highlighted by the structure of the BPH0662 genome, which contains multiple examples of chimeric mobile genetic elements. As the first complete genome of an ST2 *S. epidermidis* strain, BPH0662 provides an ideal reference genome for future bioinformatic analysis of clinical *S. epidermidis* isolates, which worldwide are dominated by the ST2 lineage. Characterisation of the BPH0662 methylome, which has been demonstrated to be representative of the ST2 lineage internationally, and construction of *E. coli* hosts that express the type I RM systems of BPH0662 will assist the development of molecular methods to bypass the restriction barrier in clinical ST2 *S. epidermidis* isolates.

## Supplementary Data

898 Supplementary File 1

## Supplementary Data

409 Supplementary File 2

## Supplementary Data

128 Supplementary File 3
