# Supplementary material for: Functional analysis of the first complete genome sequence of a multidrug resistant sequence type 2 Staphylococcus epidermidis
Source: Microb Genom. 2016 Sep 20;2(9):e000077. doi: 10.1099/mgen.0.000077 (PMC5537629; doi:10.1099/mgen.0.000077)
Supplement: Supplementary File 1 [file mgen-02-77-s001.pdf]

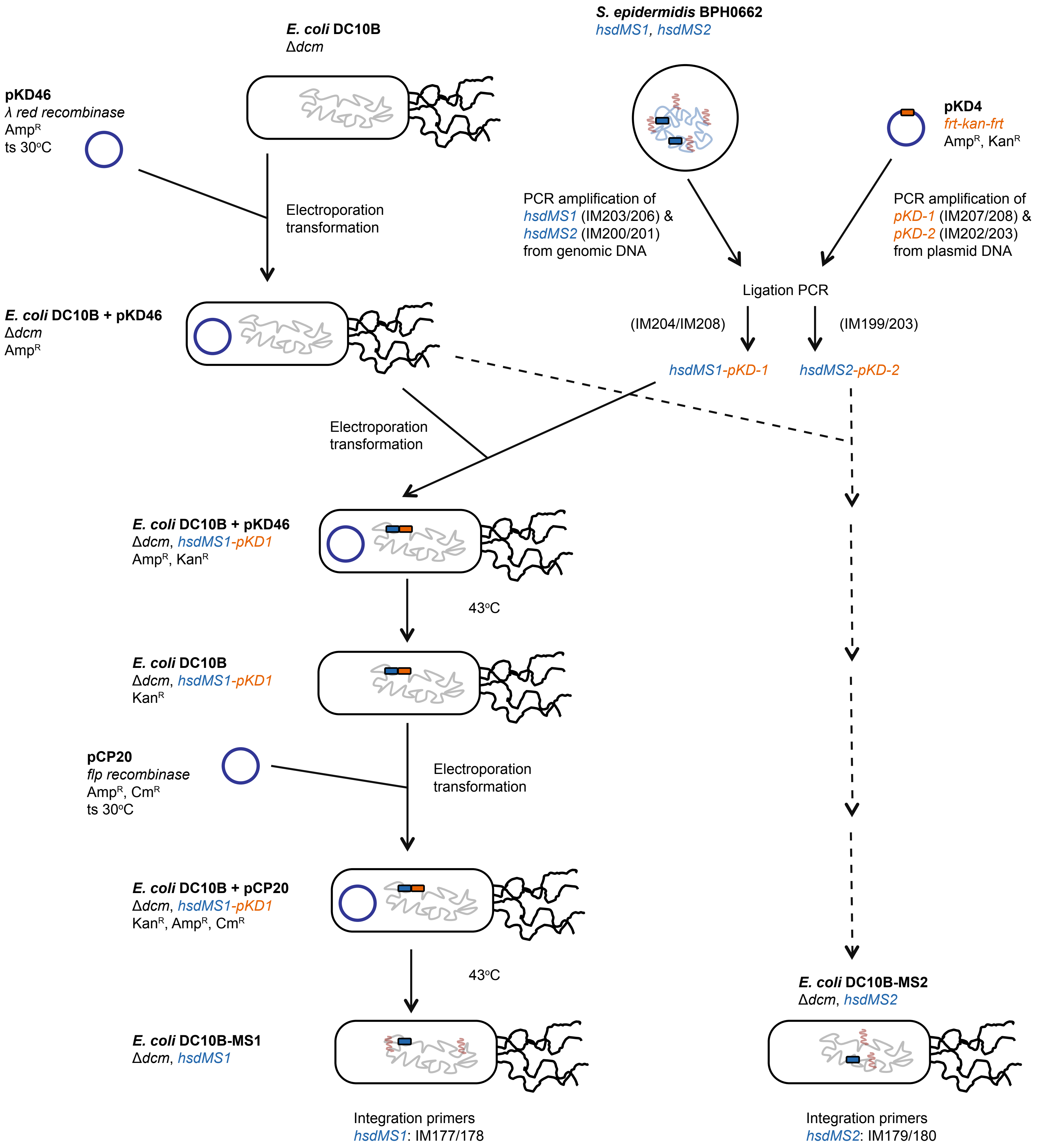

Fig. S1. Methodology for construction of *E. coli* hosts expressing the type I restriction modification systems of BPH0662. A schematic representation of the methodology for the construction of *E. coli* host strains DC10B-MS1 (which expressed the *hsdMS1* from *S. epidermidis* BPH0662) and DC10B-MS2 (which expressed the *hsdMS2* from *S. epidermidis* BPH0662). Amp^R, ampicillin resistant; ts, temperature sensitive; Kan^R, kanamycin resistant; Cm^R, chloramphenicol resistant.
