# Supplementary figures and images for: Functional analysis of the first complete genome sequence of a multidrug resistant sequence type 2 Staphylococcus epidermidis
Source: Microb Genom. 2016 Sep 20;2(9):e000077. doi: 10.1099/mgen.0.000077 (PMC5537629; doi:10.1099/mgen.0.000077)

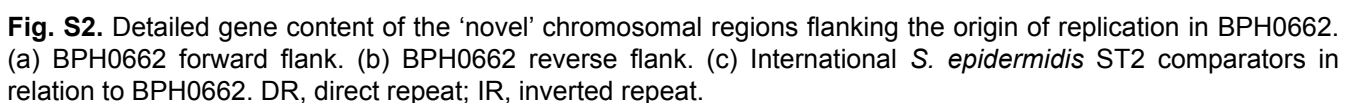

Supplement: Supplementary File 2 [file mgen-02-77-s002.pdf]
