# Supplementary material for: Functional analysis of the first complete genome sequence of a multidrug resistant sequence type 2 Staphylococcus epidermidis
Source: Microb Genom. 2016 Sep 20;2(9):e000077. doi: 10.1099/mgen.0.000077 (PMC5537629; doi:10.1099/mgen.0.000077)
Supplement: Supplementary File 3 [file mgen-02-77-s003.pdf]

Table S1. International *S. epidermidis* ST2 comparator isolates

Isolate	Sequencer	Depth	Assembler	Contigs	Genome (bp)	ok	Ns	Gaps	Min Contig Length	Avg Contig Length	Max Contig Length	N50	GC%	MLST	BioProject	BioSample	GenBank	Reference
100_SEPI	Illumina HiSeq	25x	ABYSS v. 1.3.5	122	2702163	2702163	0	0	200	22148	196587	64548	31.80	2	PRJNA267549	SAMN03196961	JWGV00000000.1	Roach et al., 2015
102_SEPI	Illumina HiSeq	30x	ABYSS v. 1.3.5	98	2677737	2677637	100	0	202	27323	190265	67289	31.80	2	PRJNA267549	SAMN03196982	JWGA00000000.1	Roach et al., 2015
1024_SEPI	Illumina HiSeq	37x	ABYSS v. 1.3.5	140	2512765	2512765	0	0	214	17948	156482	50251	31.80	2	PRJNA267549	SAMN03196988	JWFU00000000.1	Roach et al., 2015
1040_SEPI	Illumina HiSeq	52x	ABYSS v. 1.3.5	118	2603795	2603695	100	0	202	22066	196593	62363	31.90	2	PRJNA267549	SAMN03197006	JWFC00000000.1	Roach et al., 2015
1190_SEPI	Illumina HiSeq	35x	ABYSS v. 1.3.5	102	2737632	2737523	109	0	200	26839	191328	70302	31.80	2	PRJNA267549	SAMN03197150	JVZO00000000.1	Roach et al., 2015
1204_SEPI	Illumina HiSeq	31x	ABYSS v. 1.3.5	145	2491124	2491124	0	0	206	17180	159304	48890	32.00	2	PRJNA267549	SAMN03197166	JVYY00000000.1	Roach et al., 2015
596_SEPI	Illumina HiSeq	29x	ABYSS v. 1.3.5	180	2535005	2534477	528	0	218	14083	96274	31445	31.80	2	PRJNA267549	SAMN03197795	JVAT00000000.1	Roach et al., 2015
604_SEPI	Illumina HiSeq	25x	ABYSS v. 1.3.5	73	2527247	2526847	400	0	228	34619	208195	69295	32.10	2	PRJNA267549	SAMN03197804	JVAK00000000.1	Roach et al., 2015
642_SEPI	Illumina HiSeq	30x	ABYSS v. 1.3.5	85	2561559	2560757	802	0	204	30135	150058	67270	31.90	2	PRJNA267549	SAMN03197843	JUYX00000000.1	Roach et al., 2015
678_SEPI	Illumina HiSeq	109x	ABYSS v. 1.3.5	135	2682365	2682356	9	0	204	19869	189766	53486	31.90	2	PRJNA267549	SAMN03197880	JUXM00000000.1	Roach et al., 2015
767_SEPI	Illumina HiSeq	75x	ABYSS v. 1.3.5	196	2556195	2554792	1403	0	204	13041	82486	26206	31.80	2	PRJNA267549	SAMN03197976	JUTU00000000.1	Roach et al., 2015
872_SEPI	Illumina HiSeq	104x	ABYSS v. 1.3.5	149	2585892	2585892	0	0	204	17354	177645	53543	31.90	2	PRJNA267549	SAMN03198079	JUPV00000000.1	Roach et al., 2015
973_SEPI	Illumina HiSeq	41x	ABYSS v. 1.3.5	123	2707637	2707537	100	0	200	22013	177663	56080	31.80	2	PRJNA267549	SAMN03198193	JULL00000000.1	Roach et al., 2015
C10C	Illumina MiSeq	32x	CLC Genomic Workbench v. 2014	63	2540116	2540116	0	0	550	40319	283658	76639	31.80	2	PRJNA246628	SAMN02996614	JQHC00000000.1	Chong et al., 2014. *S. epidermidis* C10C genome sequence. Unpublished.
NGS-ED-1110	Illumina MiSeq	200x	SPAdes v. FEB-2015	45	2664995	2664995	0	0	1116	59222	207733	96796	31.90	2	PRJNA255947	SAMN03396286	JZUM00000000.1	Walsh et al., 2015
